# Major Adverse Cardiovascular Events after Treatment in Early-stage Breast Cancer Patients Receiving Hormone Therapy

**DOI:** 10.1038/s41598-020-57726-z

**Published:** 2020-01-29

**Authors:** Ying-Hsiang Chou, Jing-Yang Huang, Edy Kornelius, Jeng-Yuan Chiou, Chien-Ning Huang

**Affiliations:** 1 Institute of Medicine, Chung Shan Medical University, Taichung, Taiwan, ROC; 2 School of Health Policy and Management, Chung Shan Medical University, Taichung, Taiwan, ROC; 3 Department of Medical Imaging and Radiological Sciences, Chung Shan Medical University, Taichung, Taiwan, ROC; 4 Department of Radiation Oncology, Chung Shan Medical University Hospital, Taichung, Taiwan, ROC; 5 Division of Endocrinology and Metabolism, Department of Medicine, Chung Shan Medical University Hospital, Taichung, Taiwan, ROC; 6 Department of Medical Research, Chung Shan Medical University Hospital, Taichung, Taiwan, ROC

**Keywords:** Breast cancer, Oncology

## Abstract

This nationwide population-based study investigated the differences in the risks of major adverse cardiovascular events (MACEs) among patients with hormone receptor–positive early-stage breast cancer undergoing different combinations of adjuvant treatments in Taiwan. Data from the National Health Insurance Research Database (NHIRD) and Taiwan Cancer Registry (TCR) along with the national mortality data were used. Patients who underwent surgery as the first mode of treatment were divided into four groups based on the subsequent adjuvant therapy received: hormone therapy (H), hormone therapy + chemotherapy (CH), hormone therapy + radiotherapy (RH), and hormone therapy + radiotherapy + chemotherapy (CRH) groups. The risks of fatal and nonfatal MACE among the groups were examined using the inverse probability of treatment weighted hazard ratio (IPTW-HR). Adjuvant treatment, age, tumour size, and comorbidities significantly affected the risks of MACEs among the 19,007 patients analysed. For nonfatal MACEs, the IPTW-HR was significantly lower in the CH group compare to the H group (0.704, 95% confidence interval [CI]: 0.516–0.961). No significant differences in the risks for fatal MACE were observed among the four groups. The IPTW-HRs for haemorrhagic stroke in the CH group was 0.424 (95% CI: 0.188–0.957), for congestive heart failure (CHF) in the RH group was 0.260 (95% CI: 0.088–0.762), and for ischaemic heart disease in the CRH group was 0.544 (95% CI: 0.317–0.934). Increase in the adjuvant modality does not necessarily increase the nonfatal or fatal MACE risks. Cardiac health should be monitored even in patients receiving hormone therapy alone.

## Introduction

The survival of patients with breast cancer has improved with the use of aggressive screening and advances in medication and radiation techniques. For patients with hormone receptor–positive early-stage breast cancer, current guidelines even suggest extended hormone therapy for 10 years^1^. As the number of breast cancer survivors is increasing, maintaining their health is becoming a critical issue. Among older breast cancer survivors, cardiovascular diseases are the leading cause of death^2^.

The increased risk of heart disease after breast cancer treatment has been reported broadly. According to an American Heart Association report, all current adjuvant treatments that are used widely, such as chemotherapy (CT), radiotherapy (RT), hormone therapy, and targeted therapy, affect cardiovascular health. The use of tamoxifen or aromatase inhibitors (AIs) increases ischaemic heart disease risk, and RT to the left breast causes more cardiotoxicity than that to the right breast^3^. Despite these adverse impacts, in the real world, patients are often administered both these treatments, and they may even be administered other modalities with similar toxicity. Thus, in theory, the more the number of administered adjuvant treatments, the higher is the cardiovascular disease risk. However, the available information in this regard is inadequate; in addition, most studies have compared the effect of only one adjuvant treatment on cardiovascular health^4–7^.

Therefore, in this nationwide retrospective cohort population-based study, we investigated the risk of major adverse cardiovascular events (MACEs), requiring hospital admission, after breast cancer treatments in patients with hormone receptor–positive early-stage breast cancer, including those admitted in the emergency department. We then compared these risks among different adjuvant treatment groups.

## Material and Methods

### Data source

In Taiwan, under the National Health Informatics Project, Taiwanese researchers have access to more than 50 health and welfare-related datasets. All these datasets are collected, organised, and managed by the Health and Welfare Data Science Center of the Ministry of Health and Welfare (MOHW). The datasets used in this study include the National Health Insurance (NHI) Research Database (NHIRD), Taiwan Cancer Registry (TCR), and national mortality data.

NHI covers more than 99% of the country’s population; the NHIRD provides the following NHI claims data: (1) demographic and enrolment information; (2) pharmacy dispensing (outpatient and inpatient); (3) diagnosis (ambulatory, emergency, and inpatient care), coded according to the International Classification of Diseases, Ninth Revision, Clinical Modification (ICD-9-CM) system (until the year 2015); (4) procedures (radiology, endoscopy, surgery, and special examinations), coded according to the local system; (5) dental care; and (6) selected traditional Chinese medicine consultation and medication.

Since 2002, the TCR has been collecting data from hospitals by using a long form system; the registry includes detailed information on patients’ cancer stages, treatments, and follow-ups. This list includes six major cancers, namely cancers of the oral cavity and pharynx, colon and rectum, liver, lung, breast, and cervix uteri. In 2009, this list was extended to include oesophagus, stomach, prostate and bladder cancers.

The national mortality data was obtained from the household registration system, which contains birth, marital status, and death information collected by the Department of Household Registration, Ministry of the Interior (Taiwan). The cause of death is coded by MOHW based on the death certificates collected and then added into the mortality dataset.

### Identification of study group

Women newly diagnosed as having breast cancer over January 1, 2007, to December 31, 2014, were identified from the NHIRD using the ICD-9-CM code 174. Patients with stage-I and -II breast cancer who received surgery as the first mode of treatment, according to the TCR, were selected. Those diagnosed before 2010 were staged on the basis of TNM Classification of Malignant Tumours (6th edition); after 2010, the staging was based on TNM Classification of Malignant Tumours (7th edition). Patients with hormone receptor–positive early-stage breast cancer were identified based on the claims for tamoxifen or AIs from the NHIRD. Based on the treatment type, these patients were divided into hormone therapy (H), hormone therapy + CT (CH), hormone therapy + RT (RH), and hormone therapy + RT + CT (CRH) groups. The two criteria for inclusion in the study were: the first adjuvant treatment (CT, RT, and/or hormone therapy) should be started within 3 months after the surgery and CT and/or RT should be completed before the index date. The index date was set at 1 year after the patient was diagnosed with breast cancer. After the application of the exclusion criteria (specified in the subsequent section), a total of 19,007 patients were included in this study. The study was approved by the Institutional Review Board of Chung Shan Medical University Hospital, which also granted a waiver for obtaining informed consent from the patients.

### Exclusion criteria

The exclusion criteria were as follows: missing demographic data; male sex; age <18 or >80 years; history of other cancer before the index date (ICD-9-CM 140–239); death before index date; diagnosis of cardiovascular disease before the index date (ICD-9-CM 410–414 and 420–438); receipt of CT or RT after the index date; recurrence after the index date according to the TCR; never had surgery; receipt of no adjuvant treatment after surgery; no receipt of any treatment immediately before the surgery; presence of bilateral breast cancer; use of trastuzumab; missing tumour size data; and receipt of no hormone therapy. Those who ever used trastuzumab were excluded in order to narrow the study population into hormone receptor–positive Her2-negative patients. There were no molecular data regarding ER, PR, or Her2 status in the TCR until 2010; therefore, the Her2 receptor–positive patients were excluded by using this exclusion criterion instead.

### Study endpoint

The study endpoint was the occurrence of MACEs, identified by emergency visit claims or inpatient data of ICD-9-CM codes of ischaemic heart disease (410–414), CHF (402.01, 402.91, 425, and 428), acute ischaemic stroke (433–438), and intracranial haemorrhage (430–432). All patients were followed from the index date until they were censored for death, recurrence, or until December 31, 2015.

### Potential covariates

Potential covariates are comorbidities potentially related to MACE or to the general performance of the patient. Valvular heart disease (ICD-9-CM: 746.3, 747.22, 746.5), hypertension (ICD-9-CM: 401–405) diagnosed before the breast cancer diagnosis were used as the covariates related heart conditions. Diabetes mellitus (ICD-9-CM: 250), hyperlipidemia (ICD-9-CM: 272), abnormal liver function (ICD-9-CM: 570–573 and 790.4), peptic ulcer (ICD-9-CM: 531–533), abnormal renal function (ICD-9-CM: 582–586), chronic obstructive pulmonary disease (ICD-9-CM: 491, 492, and 496), mental disorder (ICD-9-CM: 290–319), rheumatic disease (ICD-9-CM: 714, 710, 710.2, 720, 696.1, and 696), thyroid disorder (ICD-9-CM: 240–245), and osteoporosis (ICD-9-CM: 733) were selected to mimick the Charlson Comorbidity Index^8^. Lifestyle covariates such as cigarette smoking status and body mass index were not included in the current study because this information was missing in more than one-third of the TCR database. Notably, the prevalence of smoking among females in Taiwan was relatively low (4.7%–5.9%) compared with that in Western countries, such as the United States (15.3% in 2015)^9,10^.

### Statistical analysis

MACE incidence was defined as the number of events fulfilling the diagnosis divided by the sum of the person-year within the follow-up interval. The Kaplan–Meier method was used to create cumulative incidence curves. Log-rank tests were used for comparison. Firstly, we used the multivariate Cox proportional hazard model to estimate the hazard ratio (HR) and 95% confidence interval (CI) by variables listed in Table 1. However, the baseline characteristics had large difference between study groups, we used inverse probability of treatment weighted hazard ratio (IPTW-HR) to identify the risk of nonfatal and fatal MACEs between 4 study groups after balance the propensity score among groups^11^. Covariates listed in Table 1 were used for calculation of propensity score by the Toolkit for Weighting and Analysis of Nonequivalent Groups (twang package in R)^12^. To balance the covariates between groups, inverse probability of weights of propensity scores was calculated (See the Supplemental File). This includes a generalized boosted model based on 5000 regression trees to define weights for optimal balance for each study group (R gbm algorithm). Weight estimates representing average effects for 4 adjuvant groups were then derived^13^. The result was considered statistically significant if *p* was <0.05. All statistics were calculated using SAS (version 9.4 for Windows; SAS Institute, Inc., Cary, NC, USA).

**Table 1:** Baseline characteristic among study population, stratified by adjuvant treatment groups.

	Adjuvant groups			
	H group	CH group	RH group	CRH group
**Type of Hormone**				
Anti-estrogens	2,468 (76.62%)	4237 (77.05%)	3041 (80.68%)	5090 (78.09%)
Aromatase inhibitors	441 (13.69%)	692 (12.58%)	423 (11.22%)	840 (12.89%)
Both	312 (9.69%)	570 (10.37%)	305 (8.09%)	588 (9.02%)
**Age**				
<45	485 (15.06%)	1268 (23.06%)	877 (23.27%)	2065 (31.68%)
45–59	1,431 (44.43%)	3166 (57.57%)	2037 (54.05%)	3600 (55.23%)
>=60	1,305 (40.52%)	1065 (19.37%)	855 (22.69%)	853 (13.09%)
**Lateral**				
Right	1,540 (47.81%)	2735 (49.74%)	1836 (48.71%)	3170 (48.63%)
Left	1,681 (52.19%)	2764 (50.26%)	1933 (51.29%)	3348 (51.37%)
**Pathological stage**				
1	2236 (69.42%)	1973 (35.88%)	3119 (82.75%)	2521 (38.68%)
2	985 (30.58%)	3526 (64.12%)	650 (17.25%)	3997 (61.32%)
**Tumour size (cm)**				
<2	2205 (68.46%)	2311 (42.03%)	3092 (82.04%)	3371 (51.72%)
2–4	858 (26.64%)	2811 (51.12%)	612 (16.24%)	2780 (42.65%)
>=4	158 (4.91%)	377 (6.86%)	65 (1.72%)	367 (5.63%)
**Co-morbidities**				
Valvular heart disease	74 (2.30%)	134 (2.44%)	93 (2.47%)	194 (2.98%)
Hypertension	1052 (32.66%)	1258 (22.88%)	824 (21.86%)	1193 (18.30%)
Diabetes mellitus	513 (15.93%)	641 (11.66%)	430 (11.41%)	642 (9.85%)
Hyperlipidemia	757 (23.5%)	952 (17.31%)	739 (19.61%)	1006 (15.43%)
Abnormal liver function	493 (15.31%)	1049 (19.08%)	543 (14.41%)	1153 (17.69%)
Peptic ulcer	469 (14.56%)	784 (14.26%)	521 (13.82%)	836 (12.83%)
Abnormal renal function	100 (3.1%)	89 (1.62%)	91 (2.41%)	99 (1.52%)
COPD	134 (4.16%)	182 (3.31%)	123 (3.26%)	198 (3.04%)
Mental disorder	810 (25.15%)	1406 (25.57%)	847 (22.47%)	1617 (24.81%)
Rheumatic disease	131 (4.07%)	185 (3.36%)	140 (3.71%)	205 (3.15%)
Thyroid disorder	238 (7.39%)	415 (7.55%)	304 (8.07%)	483 (7.41%)
Osteoporosis	248 (7.70%)	198 (3.6%)	160 (4.25%)	177 (2.72%)

H group: hormone therapy alone; CH group: chemotherapy and hormone therapy; RH group: radiotherapy and hormone therapy; CRH group: chemotherapy, radiotherapy and hormone therapy; COPD: chronic obstructive pulmonary diseas.

## Results

**Figure 1:**
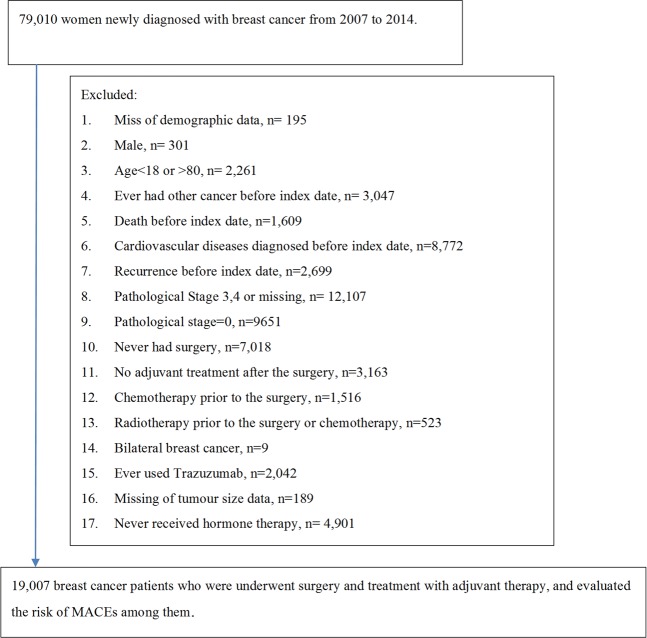
Study population selection. Patients with early-stage breast cancer receiving surgery prior to the adjuvant treatments were selected from the NHIRD and linked to the TCR and national mortality database. NHIRD: National Health Insurance Research Database; MACE: major adverse cardiovascular events.

We retrieved and analysed the data of 27,466 patients who were newly diagnosed with breast cancer from 2007 to 2014. All of them had undergone primary surgery as the first mode of treatment and had obtained histopathological confirmations of stage-I or -II disease. Patients who had not received hormone therapy or had been treated with trastuzumab were excluded. Finally, 19,007 patients were included in the study cohort (Fig. 1)*;* stage-I and -II breast cancer was noted in 9,849 (51.82%) and 9,158 (48.18%) patients, respectively. Moreover, 24.70%, 53.84%, and 21.46% of the patients were diagnosed at the age of <45, 45–60, and >60 years, respectively, and 16.95%, 28.93%, 19.83%, and 34.29% of all patients were in the H, CH, RH, and CRH groups, respectively. Approximately 75%–80% of the patients across the different treatment groups received antiestrogen (tamoxifen), whereas AIs were evenly used in 11.22%–13.69% of the patients; the remaining patients received both the drugs. In the H group, 40.52% of the patients were aged >60 years and 15.06% were aged <45 years. In the CRH group, 13.09% of the patients were aged >60 years and 31.68% were aged <45 years. The characteristics common among the patients in the study population are listed in Table 1.

The adjusted HRs (aHRs) for different variables are estimated by multiple Cox Regression and listed in Table 2. For nonfatal MACEs, the CH group had an aHR of 0.622(95% CI: 0.459–0.843). Patients aged >45 years, with hypertension, with diabetes mellitus, with abnormal renal function, and with any mental disorder had a high nonfatal MACE risk. The RH and CRH groups had a lower aHR for fatal MACEs. Patients using both antiestrogen and AIs, aged >60 years, with >2-cm-sized tumour, with hypertension, with diabetes mellitus, and with abnormal renal function had high fatal MACE risk.

**Table 2:** aHR for Nonfatal or fatal MACE.

Variables	Nonfatal MACE		Fatal MACE	
	aHR (95% C.I.)	p	aHR (95% C.I.)	p
**Adjuvant therapy**, **ref: H group**				
CH group	**0**.**622** (**0**.**459–0**.**843)**	**0**.**002**	0.883 (0.689–1.131)	0.330
RH group	0.831 (0.593–1.164)	0.286	**0**.**609** (**0**.**430–0**.**862)**	**0**.**005**
CRH group	**0**.**749** (**0**.**548–1**.**024)**	**0**.**070**	**0**.**712** (**0**.**545–0**.**932)**	**0**.**013**
**Type of Hormone**, **ref: Anti-estrogens**				
Aromatase inhibitors	0.767 (0.517–1.138)	0.189	1.023 (0.736–1.420)	0.900
Both	1.279 (0.935–1.750)	0.124	**1**.**749** (**1**.**371–2**.**231)**	**<0**.**001**
**Age**, **ref: <45**				
45–59	**1**.**777** (**1**.**179–2**.**679)**	**0**.**006**	1.097 (0.859–1.401)	0.467
>=60	**4**.**795** (**3**.**094–7**.**430)**	**<0**.**001**	**1**.**854** (**1**.**383–2**.**486)**	**<0**.**001**
**Laterality**, **ref: Right**				
Left	0.953 (0.769–1.180)	0.672	1.074 (0.904–1.275)	0.424
**Pathological stage**, **ref:** 1				
2	1.041 (0.739–1.467)	0.830	**1**.**321** (**0**.**999–1**.**746)**	**0**.**050**
**Tumour size** (**cm)**, **ref:** <2				
2–4	1.299 (0.933–1.808)	0.121	**1**.**673** (**1**.**285–2**.**178)**	**<0**.**001**
>=4	1.380 (0.826–2.305)	0.221	**2**.**648** (**1**.**871–3**.**749)**	**<0**.**001**
**Co-morbidities**				
Valvular heart disease	1.519 (0.896–2.575)	0.121	0.893 (0.502–1.591)	0.714
Hypertension	**2**.**140** (**1**.**667–2**.**748)**	**<0**.**001**	**1**.**307** (**1**.**058–1**.**615)**	**0**.**013**
Diabetes mellitus	**1**.**586** (**1**.**211–2**.**077)**	**0**.**001**	**1**.**509** (**1**.**179–1**.**931)**	**0**.**001**
Hyperlipidemia	0.831 (0.637–1.084)	0.173	**0**.**720** (**0**.**562–0**.**923)**	**0**.**009**
Abnormal liver function	0.990 (0.750–1.307)	0.948	1.021 (0.815–1.279)	0.867
Peptic ulcer	1.026 (0.769–1.369)	0.871	1.003 (0.784–1.283)	0.983
Abnormal renal function	**2**.**476** (**1**.**585–3**.**868)**	**<0**.**001**	**2**.**393** (**1**.**583–3**.**618)**	**<0**.**001**
COPD	1.140 (0.727–1.788)	0.580	0.917 (0.595–1.413)	0.708
Mental disorder	**1**.**463** (**1**.**161–1**.**843)**	**0**.**001**	1.131 (0.929–1.376)	0.221
Rheumatic disease	1.378 (0.864–2.199)	0.179	1.321 (0.881–1.980)	0.179
Thyroid disorder	1.023 (0.686–1.523)	0.918	1.049 (0.752–1.463)	0.791
Osteoporosis	0.688 (0.429–1.102)	0.120	1.050 (0.727–1.516)	0.807

aHR: adjusted hazard ratio; MACE: major cardiovascular events, COPD: chronic obstructive pulmonary disease, H group: hormone therapy alone; CH group: chemotherapy and hormone therapy; RH group: radiotherapy and hormone therapy; CRH group: chemotherapy, radiotherapy and hormone therapy. aHR adjusted for adjuvant therapy for breast cancer, type of hormone therapy, age group, lateral of cancer, pathological stage, tumour size and co-morbidities.

**Figure 2:**
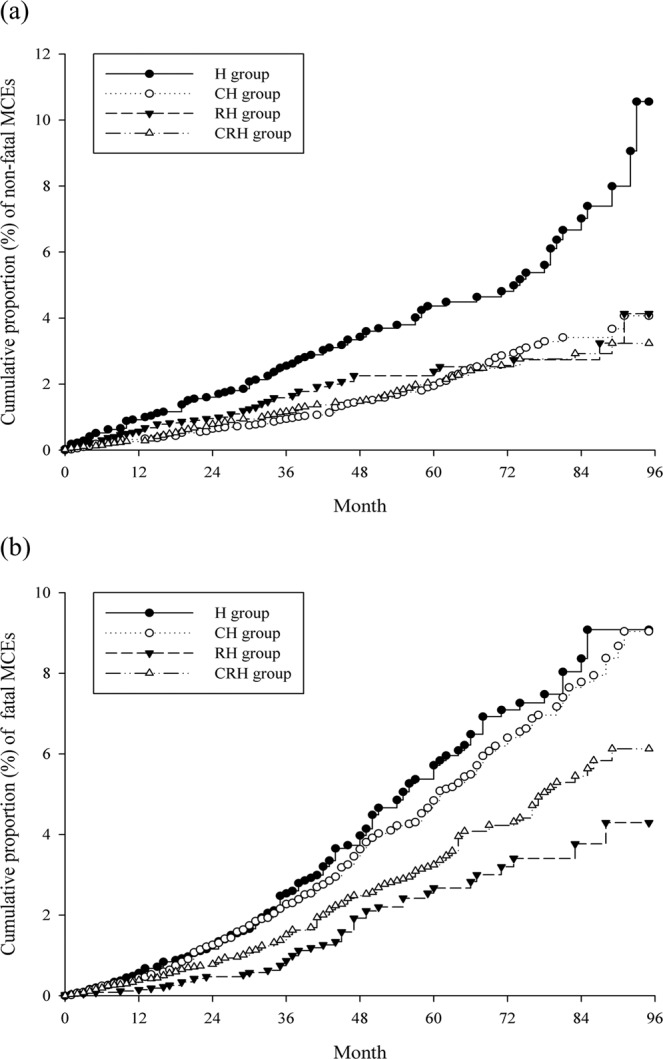
Kaplan–Meier curves of cumulative proportion of (**a**) nonfatal MACEs (log-rank p < 0.0001) (**b**) fatal MACEs. MACE: major adverse cardiovascular events. H group: hormone therapy alone; CH group: chemotherapy and hormone therapy; RH group: radiotherapy and hormone therapy; CRH group: chemotherapy, radiotherapy and hormone therapy.

**Figure 3:**
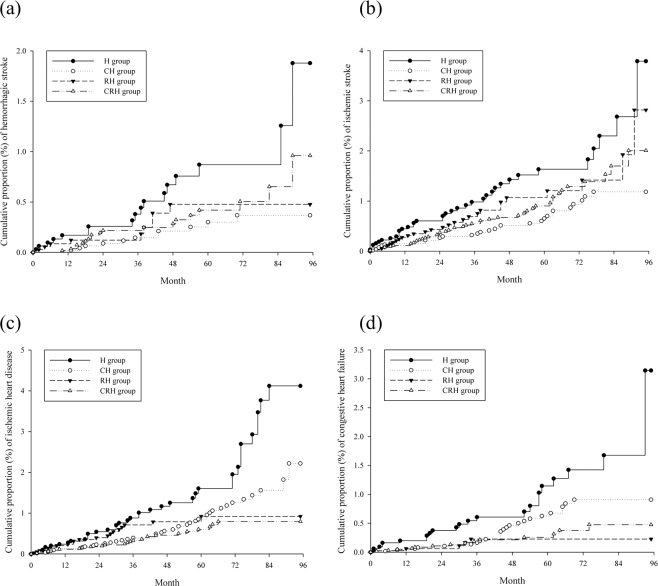
Kaplan–Meier curves of cumulative proportion of (**a**) haemorrhagic stroke, (**b**) ischaemic stroke; (**c**) ischaemic heart disease; (**d**) congestive heart failure. H group: hormone therapy alone; CH group: chemotherapy and hormone therapy; RH group: radiotherapy and hormone therapy; CRH group: chemotherapy, radiotherapy and hormone therapy.

The absolute incidence rates and IPTW-HRs are lists in Table 3. In total, 336 nonfatal MACE events were noted; the incidence rates were 7.79, 3.60, 4.21, and 3.37 per 10,000 person-months (PMs) in the H, CH, RH, and CRH groups, respectively. The IPTW-HR was significantly lower in the CH group (0.704, 95% CI: 0.516–0.961). Moreover, 522 fatal MACEs were noted; the incidence rate were 8.70, 8.28, 3.55, and 5.48 per 10,000 PMs in the H, CH, RH, and CRH groups, respectively. No significant differences in the incidence of fatal MACEs were noted between four adjuvant treatment groups. The cumulative proportions of nonfatal and fatal MACEs are show in Fig. 2. Moreover, we noted 56, 142, 126, and 67 events of haemorrhagic stroke, ischaemic stroke, ischaemic heart disease, and CHF events, respectively. For haemorrhagic stroke, the CH group showed a significant IPTW-HR (0.424, 95% CI: 0.188–0.957) compared to the H group. For ischaemic heart disease, the CRH group showed a significant IPTW-HR (0.544, 95% CI: 0.317–0.934) compared to the H group. For CHF, the RH group showed a significant IPTW-HR (0.260, 95% CI: 0.088–0.762) compared to the H group. No significant difference among treatment was noted for ischaemic stroke (Table 4 and Fig. 3).

**Table 3:** Risk of MACEs and IPTW-HR.

	Person months (PM)	Events	Incidence rate (per 10000 PMs)	IPTW-HR (95% C.I.)	p
**For nonfatal MACEs**					
H group	124,581	97	7.79	Reference	
CH group	258,687	93	3.60	**0**.**704 (0**.**516–0**.**961)**	**0**.**027**
RH group	130,632	55	4.21	0.913 (0.617–1.350)	0.662
CRH group	269,730	91	3.37	0.786 (0.572–1.079)	0.137
**For fatal MACEs**					
H group	126382	110	8.70	Reference	
CH group	260849	216	8.28	1.047 (0.798–1.374)	0.753
RH group	132231	47	3.55	0.783 (0.511–1.199)	0.264
CRH group	271992	149	5.48	0.800 (0.600–1.067)	0.129

H group: hormone therapy alone; CH group: chemotherapy and hormone therapy; RH group: radiotherapy and hormone therapy; CRH group: chemotherapy, radiotherapy and hormone therapy; MACE: major adverse cardiovascular events; IPTW-HR: inverse probability of treatment weighted hazard ratio, the propensity score was estimated as average treatment effect in the entire cohort. The predictors of the propensity score were type of hormone therapy, age group, and laterality of cancer, pathological stage, tumour size and co-morbidities.

**Table 4:** Risk of different MACEs.

	Person months (PM)	Events	Incidence rate, per 10000 PMs	IPTW-HR (95% C.I.)	p
**For haemorrhagic stroke**					
H group	126147	17	1.35	Reference	
CH group	260601	11	0.42	**0**.**424** (**0**.**188–0**.**957)**	**0**.**038**
RH group	131971	9	0.68	1.185 (0.475–2.958)	0.729
CRH group	271574	19	0.70	0.809 (0.396–1.651)	0.572
**For ischaemic stroke**					
H group	125790	37	2.94	Reference	
CH group	260098	32	1.23	0.736 (0.443–1.222)	0.239
RH group	131488	27	2.05	1.530 (0.862–2.714)	0.146
CRH group	270961	46	1.70	1.146 (0.717–1.829)	0.580
**For ischaemic heart disease**					
H group	125593	39	3.11	Reference	
CH group	259916	41	1.58	0.755 (0.470–1.213)	0.248
RH group	131635	20	1.52	0.614 (0.343–1.097)	0.100
CRH group	271210	26	0.96	**0**.**544** (**0**.**317–0**.**934)**	**0**.**027**
**For congestive heart failure**					
H group	125942	23	1.83	Reference	
CH group	260338	25	0.96	0.751 (0.400–1.410)	0.379
RH group	132072	5	0.38	**0**.**260** (**0**.**088–0**.**762)**	**0**.**014**
CRH group	271776	14	0.52	**0**.**541** (**0**.**252–1**.**163)**	**0**.**115**

H group: hormone therapy alone; CH group: chemotherapy and hormone therapy; RH group: radiotherapy and hormone therapy; CRH group: chemotherapy, radiotherapy and hormone therapy; MACE: major adverse cardiovascular events; IPTW-HR: inverse probability of treatment weighted hazard ratio, the propensity score was estimated as average treatment effect in the entire cohort. The predictors of the propensity score were type of hormone therapy, age group, and laterality of cancer, pathological stage, tumour size and co-morbidities.

After stratification according to the cancer stage, the CH and CRH group patients diagnosed as having stage-II breast cancer demonstrated significantly lower nonfatal MACE risk (IPTW-HR 0.514 and 0.641 respectively) compared with the H group. Regarding fatal MACEs, patients with pathological stage-II breast cancer in the CH group had a significantly lower IPTW-HR(0.729, 95% CI: 0.533–0.997); and those in the CRH group patients also had a significantly lower IPTW-HR (0.598, 95% CI: 0.427–0.837) (Table 5).

**Table 5:** IPTW-HR (95% C.I.) for MACEs stratified by pathological stage.

	Pathological Stage			
	1		2	
	IPTW-HR (95% C.I.)	p	IPTW-HR (95% C.I.)	p
**For nonfatal MACEs**				
H group	Reference		Reference	
CH group	0.737 (0.465–1.170)	0.196	**0**.**514** (**0**.**343–0**.**770)**	**0**.**001**
RH group	0.816 (0.536–1.243)	0.349	0.848 (0.471–1.527)	0.595
CRH group	0.854 (0.534–1.364)	0.520	**0**.**641** (**0**.**421–0**.**976)**	**0**.**038**
**For fatal MACEs**				
H group	Reference		Reference	
CH group	1.260 (0.835–1.900)	0.274	**0**.**729** (**0**.**533–0**.**997)**	**0**.**047**
RH group	**0**.**668** (**0**.**421–1**.**061)**	**0**.**087**	0.645 (0.376–1.109)	0.112
CRH group	0.971 (0.612–1.541)	0.908	**0**.**598** (**0**.**427–0**.**837)**	**0**.**003**

IPTW-HR: inverse probability of treatment weighted hazard ratio; H group: hormone therapy alone; CH group: chemotherapy and hormone therapy; RH group: radiotherapy and hormone therapy; CRH group: chemotherapy, radiotherapy and hormone therapy; MACE: major adverse cardiovascular events.

## Discussion

In this study, we analysed the real-world data regarding the absolute incidence rates of fatal and nonfatal MACEs in patients with breast cancer receiving different adjuvant treatments. Although the relationship between cardiotoxicity and CT or RT appears to be well-documented, understanding the actual risks involved with the relevant treatments is crucial. In the present study, the H group had a relatively greater risk of nonfatal MACE compared to the remaining three groups. However, cardiovascular disease-related mortality did not seem to be affected by the adjuvant treatments. Moreover, the MACE risk, in general, did not demonstrate significant differences across the groups in this study. This result is contradictory to that of numerous studies, which reported that RT and CT cause more cardiotoxicity. Confounding indicators, such as age and comorbidities, may partly explain this result; however, even after adjustment for age and propensity score, the observed trend persisted. In other words, even patients receiving hormone therapy alone as adjuvant treatment after surgery are not protected from MACEs.

In our study population, the patients belonging to the H group were older and presented with multiple comorbidities, such as hypertension, diabetes mellitus, and hyperlipidemia. Antiestrogen treatment was evenly used in 76%–80% of the patients across all the groups; 40% of these patients in the H group were >60 years old. The Taiwan NHI does not cover the use of AIs in node-negative patients; hence, the use of tamoxifen was preferred over AI. CT was administered to 70% of the patients aged <45 years and 47% of those aged <60 years. This decrease in use was also seen with RT—the decline was from 62.7% in patients aged <45 years to 41.9% in those aged >60 years. These numbers adequately correlated with those reported in the annual report of the TCR and published by the MOHW, Taiwan. According to the 2017 report, 60.19% and 51.81% of patients with breast cancer received CT and RT, respectively. Patients with stage-I breast cancer tended to be in the H or RH treatment group.

Consistent with some previously published population-based studies, 17.86% of the patients with stage-II breast cancer did not receive postsurgical CT in the current study. Researchers found that older patients tended to receive less aggressive treatments, possibly hindering their survival outcomes^14^. A study from the Netherlands reported that only 53% of the older patients received adjuvant systemic treatment whereas, among the younger patients, 79% received adjuvant therapy^15^. A comprehensive evaluation system is needed for treatment consultations among special populations.

Tamoxifen and AIs have different effects on cardiac health in patients with breast cancer. Tamoxifen has demonstrated favourable cardiovascular effects such as a reduction in total and low-density lipoprotein cholesterol (LDL-C) levels, an increase in high-density lipoprotein cholesterol levels, and a decrease in fibrinogen level^16–18^. A meta-analysis comparing tamoxifen with AIs showed that AIs were associated with a 19% increase in cardiovascular events, which may be due to the cardio-protective effect of tamoxifen^4^. However, whether tamoxifen increases the thromboembolism risk remains inconclusive^5^. In an observational study from Taiwan, female patients with breast cancer who developed acute myocardial infarction (AMI) were 1.71 times more likely to receive tamoxifen compared with those who did not develop AMI^19^. In the current study, more than 75% of the total cohort used tamoxifen in hormone therapy. The risks of fatal MACE were significantly related to the use of both antiestrogen and AIs; age >60 years; tumour size >2 cm; and presence of hypertension, diabetes mellitus, and abnormal renal function.

Certain CTs are known for their cardiotoxicity^3^. For instance, anthracycline can cause a 4% decline in left ventricular ejection fraction (LVEF) even after 3 years of exposure^20^. In few cases, antimetabolite drugs, such as 5-fluorouracil, are related to myocardial infarction, heart failure, and arrhythmias^21^. A matched cohort study from Canada examining the risk of cardiovascular hospitalisation and heart failure demonstrated that the10-year incidence of cardiovascular disease hospitalisation was 10.8% for patients with breast cancer; moreover, 37% of their study population received anthracyclines and/or trastuzumab and the relative rate of cardiovascular disease was higher compared to their age-matched subjects^22^. In our study, we excluded those who received trastuzumab to simplify the division of the patients into four adjuvant groups that represented the majority of the breast cancer population. Neither the H nor the CH group demonstrated an increasing trend in the incidence of MACE.

Darby *et al*. concluded that post-RT coronary event risk has a dose-response relationship wherein, a 7.4% increase was noted with every 1-Gy increase in dose^23^. In a recent meta-analysis, Taylor *et al*. reported that all-cause mortality was increased by radiotherapy (RR, 1.5), predominantly due to cardiac disease (particularly ischaemic heart disease); the estimated absolute risks from modern breast irradiation for cardiac morality were 1% for smokers and 0.3% for nonsmokers^7^. These conclusions are not consistent with those of other studies. In the BCIRG-001 study, patients who received uniform CT followed by RT did not show an increased ischaemic heart disease risk compared to those who did not receive the treatments^24^. In the present study, the incidence of MACE did not differ significantly between the H, RH, and CRH groups.

Few articles have discussed the crude incidence rates of cardiotoxicity when multiple adjuvant treatments are administered. The Danish Breast Cancer Cooperative Group determined the heart disease incidence rate ratios by comparing left-sided with right-sided cancer (IRR, LvR)^25^. They reported that the IRR, LvR was increased to 1.32 (95% CI: 1.02–1.70, *p* = 0.03) with anthracycline, and this value did not differ significantly between the combined RT and the hormone therapy. Kim *et al*. evaluated the prevalence of cardiovascular events including AMI, CHF, and stroke in patients with breast cancer treated with concomitant RT and CT^25^. In their study, left-side RT plus a cumulative dose of doxorubicin ( ≥ 250 mg/m^2^) were independent risk factors for both types of heart failure, AMI and stroke. The impact of hormone therapy was not addressed in their study^26^. Hooning *et al*. reported long-term cardiovascular disease and stroke risk in 10-year survivors of breast cancer. The standardised incidence ratios (SIRs; 95% CIs) of myocardial infarction were 1.33 (1.14–1.55), 1.36 (0.83–2.10), 1.38 (0.91–2.02), and 1.19 (0.61–2.08), respectively for patients who received only RT, RT + CT, RT+ hormone therapy, and RT + CT+ hormone therapy^27^. The SIR was lowest in patients who received all three adjuvant treatments (as was seen in the present study). The patterns were the same for angina pectoris, but not for CHF. They reported that the significant SIRs for heart failure were 1.23, 3.48, 1.86, and 2.66 in patients who received only RT, RT + CT, RT+ hormone therapy, and RT + CT+ hormone therapy, respectively. In the current study, the risk of CHF was significantly low in the RH group. The SIRs for stroke (90% ischaemic) were 1.31 and 0.83 in patients who received RT+ hormone therapy and RT + CT+ hormone therapy, respectively, which indicated that receiving additional adjuvant modalities did not increase the risk of stroke^28^. A Swedish study showed that patients with breast cancer had a higher SIR for ischaemic stroke, but not for haemorrhagic stroke, after treatment when compared to the normal population. Moreover, the SIR declined with the follow-up time; the values were 1.5, 1.2, 1.1 and 1.1 for <6 months, 6–12 months, 1–5, and 5–10 years of follow-up time, respectively^29^. In the current study, the CH group showed a significantly lower IPTW-HR for haemorrhagic stroke, but not ischaemic stroke, when compared with the H group. While the studies by Hoonings *et al*. focused on patients who survived for longer than 10 years and excluded all events that happened within the 10 years after treatment, our study focused on patients who were undergoing hormone treatment 5–10 years after the treatment.

MACE, a composite endpoint frequently used in cardiovascular research, is less commonly used in oncology studies. Romond *et al*. predicted the 5-year probability of heart failure or cardiac death in a model using only age and baseline left ventricular ejection fraction (LVEF)^30^. Abdel-Qadir *et al*. had developed a prediction model for MACE in patients with early-stage breast cancer. By incorporating age and underlying comorbidities such as hypertension, diabetes, ischaemic heart disease, atrial fibrillation, heart failure, cerebrovascular disease, peripheral vascular disease, chronic obstructive pulmonary disease, and chronic kidney disease into the scoring system, a good agreement between the predicted and observed incidences of MACE has been reported^31^. However, none of the treatment-related factors were considered in the aforementioned prediction models for MACE risk after treatment, despite the American Society of Clinical Oncology suggesting that patients receiving anthracycline and radiotherapy or trastuzumab have an increased risk of developing cardiac dysfunction and should be closely monitored^32^. In the present study, we reported the risk of fatal and nonfatal MACE after early-stage breast cancer treatment. Our study results suggest that the medical team must practice caution even for patients who are undergoing hormone therapy alone, particularly in the field of cardio-oncology.

Our study answers some clinically relevant questions. First, the exact cardiotoxicity after treatment, particularly in patients who required admission and intervention, was determined. Second, the differences between different adjuvant treatment groups were studied, thus providing essential oncological and cardiological information. However, this study has some limitations. First, due to the nature of population-based study, information regarding treatment details and compliance are not available. We only included patients prescribed with hormone therapy longer than 3 months. Despite including important clinical characteristics and covariables, residual confounding cannot be completely excluded as patients in the H group were older and having more comorbidities. By using the IPTW method, we believe that the balance among four groups are reached. Second, the follow-up period of the study was short. Cardiotoxicity from RT extends to >10 years after treatment, and after treatment, the effect of CT decreases rapidly^3^. In our cohort, the patients were undergoing 5–10 years of hormone therapy. Thus, long-term follow-up studies extending up to 10–15 or even 15–20 years are warranted. Third, certain important variables were lacking because the study was based on the TCR and the reimbursement databases (NHIRD). For example, data regarding the ER, PR, and Her2 status were only collected from the year 2010. The completeness and accuracy of these databases are of great concern in most population-based studies. After complex procedures of auditing, the completeness of the TCR was reported to be 97.6% in 2006 and 97.7% in 2011^33^. However, in the case of the NHIRD, the data are mainly entered for the purpose of reimbursement; thus, further verification is warranted. For example, the type of hormone therapy prescribed was based on the NHIRD and verified in the TCR. Nonetheless, we chose a robust endpoint (MACEs requiring admission) to improve the reliability of our study.

## Conclusion

The risk of fatal or nonfatal MACE did not increase with the increase in the adjuvant modalities. In addition to these adjuvant treatments, several factors, such as age, cancer stage, and comorbidities, can increase these risks. Furthermore, caution related to MACEs is warranted, even in patients receiving hormone therapy only.

## Supplementary information

Supplementary information.
