# Supplementary material for: Major Adverse Cardiovascular Events after Treatment in Early-stage Breast Cancer Patients Receiving Hormone Therapy
Source: Sci Rep. 2020 Jan 29;10:1408. doi: 10.1038/s41598-020-57726-z (PMC6989448; doi:10.1038/s41598-020-57726-z)
Supplement: Supplementary file 1 — Supplementary information. [file 41598_2020_57726_MOESM1_ESM.pdf]

Major Adverse Cardiovascular Events after Treatment in Early-stage Breast Cancer Patients Receiving Hormone Therapy

Ying-Hsiang Chou, Jing-Yang Huang, Edy Kornelius, Jeng-Yuan Chiou, Chien-Ning Huang*

***Corresponding:**
Chien-Ning Huang, MD, PhD (cshy049@csh.org.tw)

Supplementary Information:
Supplementary Table 1：Balance diagnosis after propensity score weighting
Supplementary Table 2：The diagnosis of propensity score after IPTW

Supplementary Figure 1：Balance measure in each iteration.
Supplementary Figure 2：Absolute standardized difference (maximum pairwise) before and after IPTW

Supplementary Table 1 Balance diagnosis after propensity score weighting

Stop method	Max. absolute standardized difference	Max. KS
Unweight	0.901	0.447
es.mean	0.028	0.013
ks.mean	0.024	0.011

The Max. absolute standardized differences were less than 0.1 in either es.mean or ks.mean stop method, and Max. KS also indicate the co-variates were balance after weighting.

Supplementary Table 2 The diagnosis of propensity score after IPTW

	Max. absolute standardized difference		
Variable	Stop method: Unweight	Stop method: es.mean	Stop method: ks.mean
Age group	0.543	0.017	0.015
Lateral	0.041	0.009	0.004
Pathological stage	0.901	0.025	0.022
Tumor size	0.769	0.003	0.005
Valvular heart disease	0.036	0.015	0.011
Hypertension	0.317	0.008	0.006
Diabetes mellitus	0.200	0.013	0.008
Hyperlipidemia	0.202	0.013	0.009
Abnormal liver function	0.114	0.028	0.024
Peptic ulcer	0.089	0.008	0.010
Abnormal renal function	0.141	0.012	0.009
COPD	0.084	0.007	0.009
Mental disorder	0.059	0.021	0.017
Rheumatic disease	0.028	0.012	0.008
Thyroid disorder	0.049	0.006	0.008
Osteoporosis	0.192	0.021	0.022

The Max. absolute standardized differences were less than 0.1 in all co-variate variables.

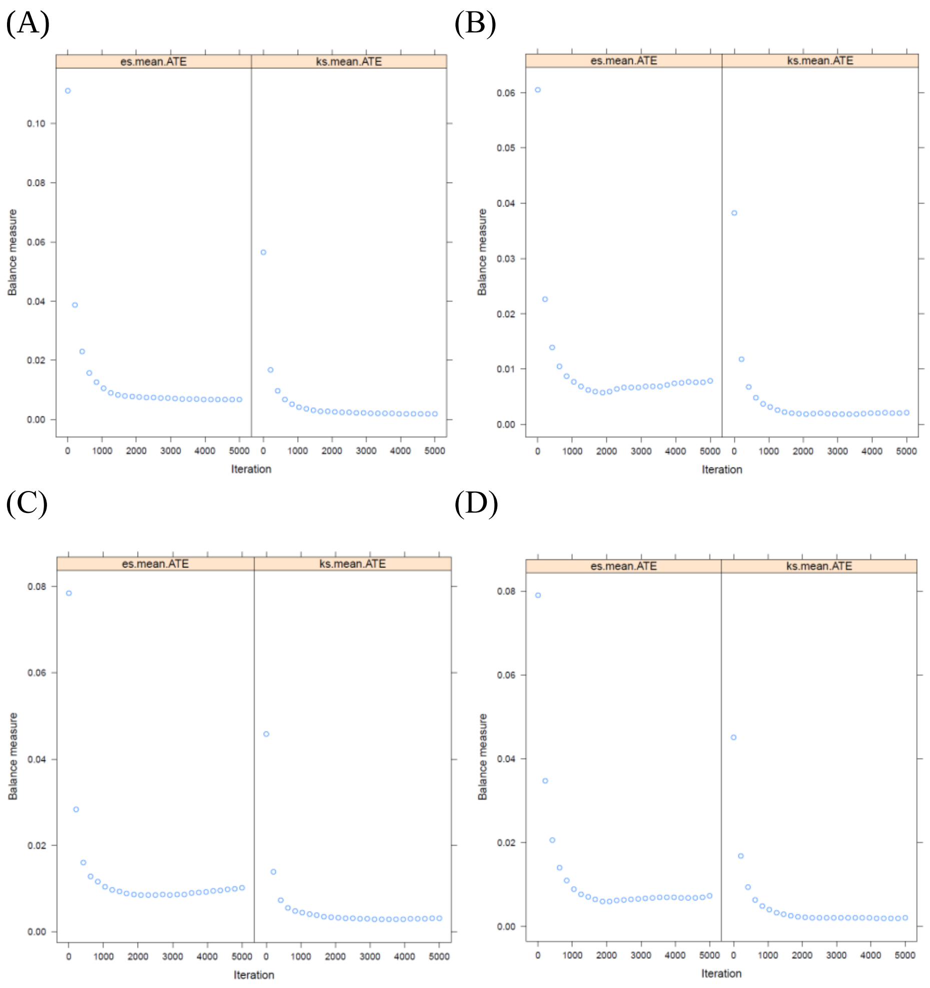

Supplementary Figure 1. Balance measure in each iteration.

(A) H group against others. (B) CH group against others. (C) RH group against others. (D) CRH group against others

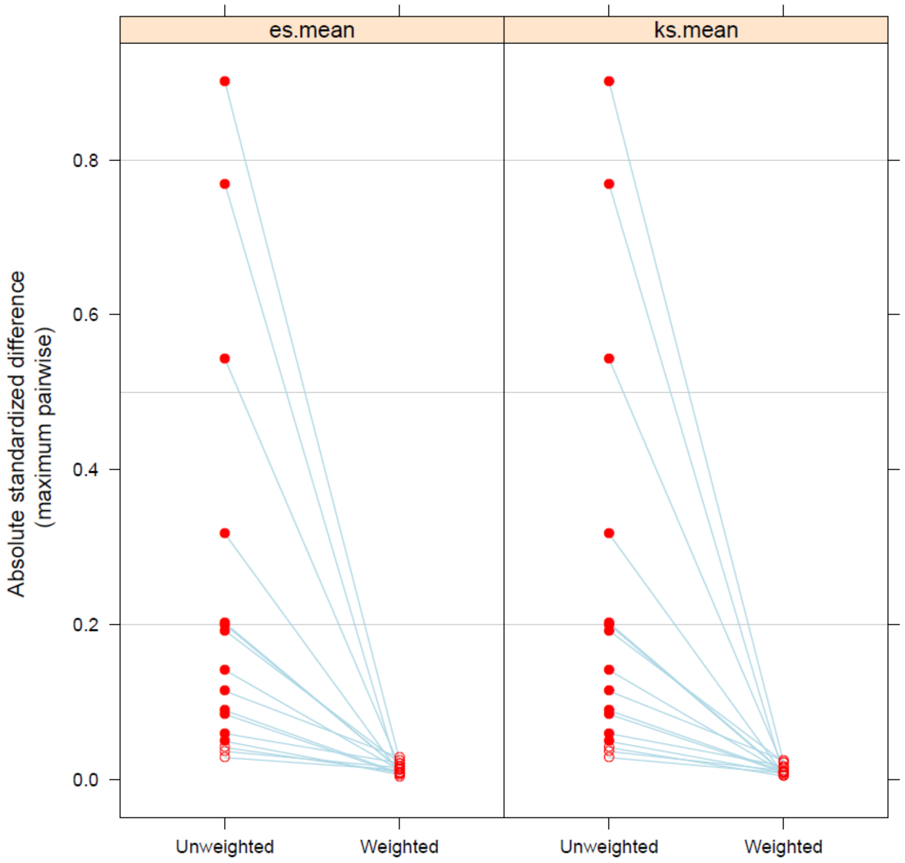

Supplementary Figure 2. Absolute standardized difference (maximum pairwise) before and after IPTW
